# The Effect of Activity Participation in Middle-Aged and Older People on the Trajectory of Depression in Later Life: National Cohort Study

**DOI:** 10.2196/44682

**Published:** 2023-03-23

**Authors:** Yan Gao, Zhihao Jia, Liangyu Zhao, Suyue Han

**Affiliations:** 1 School of Physical Education Shandong University Jinan, Shandong China

**Keywords:** activity participation, middle-aged and older people, depression, cohort study, developmental

## Abstract

**Background:**

More activity participation is an important means of handling depression and promoting positive aging, but the impact of changes in activity participation on the developmental trajectory of depression has not been fully studied.

**Objective:**

The purpose of this study is to study the relationship between current activity participation and depression in middle-aged and older people (≥45 years old) and the relationship between activity participation and the developmental trajectory of depression in later life in China.

**Methods:**

This study used data from the China Health and Retirement Longitudinal Study (CHARLS) across 7 years and included a total of 4818 middle-aged and older people (≥45 years old). Controlling for relevant control variables, the latent growth curve model and the cross-lag model were used to assess the effect of changes in activity participation on the depression trajectory in later life and the main lag relationship between activity participation and depression. Activity participation as well as depression were measured using the self-reported activity and health status based on the CHARLS questionnaire.

**Results:**

Among the 4818 respondents, the mean values of physical activity participation, social leisure activity participation, and depression ranged from 76.98 (SD 15.16) to 83.95 (SD 5.72), from 7.43 (SD 8.67) to 9.25 (SD 10.16), and from 7.61 (SD 5.72) to 8.82 (SD 6.51), respectively. Our findings revealed that activity participation could be related to depression. Physical activity participation predicted initial depression (*β*=–0.631, *P*<.001) and its trajectory (*β*=0.461, *P*<.001). However, social leisure activity participation predicted initial depression (*β*=–0.223, *P*<.001) but did not predict its trajectory (*β*=0.067, *P*=.159). Finally, cross-lag regression analysis further demonstrated the predictive effect of activity participation on depression.

**Conclusions:**

This study demonstrates the prediction of activity participation for future depression in the Chinese middle-aged and older populations. The data showed that activity participation is significantly associated with changes in depression and future depression among middle-aged and older people in China. The Chinese government should encourage middle-aged and older people to participate in various activities, which can effectively prevent the aggravation of depression and also have a positive significance for positive aging.

## Introduction

Better activity participation not only has a positive impact on the physical and mental health of middle-aged and older people [1-3] but also improves the health-related quality of life (HRQoL) [4,5]. Specifically, active participation in various activities can provide middle-aged and older people with a series of positive feelings so that they can better cope with the pressure of daily life and maintain good social interaction. This will make them feel happier and more satisfied with their lives, which will further result in improved HRQoL and mental health [3].

At present, activity participation has become a hot topic in depression research. If the elderly cannot adapt to changes brought about by physical function (PF) decline or the change in social roles (eg, changes in activity participation), anxiety and depression will be an inevitable consequence [6]. Depression is a common mental disorder. Worldwide, an estimated 5% of adults have depression. According to statistics, after the COVID-19 pandemic, there were about 374 million cases of anxiety (4802 cases per 100,000 people) and 246 million cases of major depression (3153 cases per 100,000 people), up by 26% and 28%, respectively, compared to the previous years [7]. Therefore, mental health problems have become a big problem worldwide. At the same time, the prevalence of depression in China is also a big issue. It is estimated that the prevalence of depression in the elderly in China is as high as 23.6%, and its prevalence will increase not only with age [8,9] but also with a trend of “rejuvenation” [10]. It is clear that depression in the middle-aged and the older population has become a major public health problem in China. Exploring the developmental trajectory of depression in the middle-aged and older populations and its relationship with related factors is of great significance for actively coping with healthy aging.

Although some studies have shown that activity participation levels in middle-aged and older adults increase or stabilize over time [11,12], in general, the academic community generally believes that it becomes increasingly difficult for middle-aged and older people to maintain active participation as they grow older [13,14]. The concept of life cycle is widely used. In psychology, it mainly refers to the life cycle of people and the life cycle of the family and also refers to the process of birth, growth, aging, illness, and death. Life cycle theory holds that after middle-aged people enter old age, their physiological and psychological processes experience a series of changes [14], which leads to changes in activity participation [6]. In a nutshell, the activity participation of the elderly (eg, leisure activities) gradually becomes simpler and decreases over time [11,14]. Numerous studies have shown that older adults generally have lower levels of social activity participation, and this lower level may further decrease with age [15-17]. Approximately 39.0% of respondents showed low social activity engagement at baseline and decreased engagement within 10 years [15]. Especially among the elderly in China, they spend more time sedentary than the young [18]. In addition, life cycle theory [14] believes that when entering the final stage of the life cycle, both physical and mental functions decline sharply, suggesting that the trends in activity participation and depression should be nonlinear.

Currently, there are a large number of studies focusing on the relationship between activity participation and depression. Activity theory is commonly used to explain the relationship between social participation and mental health in older adults. Activity theory believes that middle-aged and older people can establish new roles to cope with the loss of their original roles so as to adapt to society and obtain psychological satisfaction [19]. In other words, older people can establish new roles through activity participation to alleviate the depressive symptoms caused by role changes [6]. This theory is also supported by the results of some clinical studies, which suggest that activity participation can prevent depression [20] and is becoming an effective treatment for depression [21]. This shows that leisure activities are actually a protective factor for depression in the elderly and that participation in activities may reduce the rate of depression [22]. Additionally, studies have shown that changes in PF and changes in depression are reciprocal [23-25]. Given the direct link between activity participation and PF, activity participation changes may also be associated with changes in depression. Therefore, changes in activity participation in middle-aged and older adults may predict changes in depression. Finally, behavioral activation (BA) theory suggests that engaging in activities that make individuals feel pleasure improves depression and promotes activity participation after depression improves [26]. This suggests that there may be a temporal sequence between activity participation and depression. Therefore, this study further determines the temporal order of the relationship between activity participation and depression.

Previous studies have often included activity participation as a time-invariant dependent variable or a simple control variable and have simply verified the correlation between the initial levels of the 2 variables [22,27]. The effect of changes in activity participation on depression was rarely considered, and this led to a lack of discussion of the relationship between the initial levels of the 2 variables and the rate of change. Therefore, this study selected data from the China Health and Retirement Longitudinal Study (CHARLS) spanning 7 years using a latent growth curve model (LGCM) and a cross-lag model to explore the developmental trajectories of activity participation and depression and the patterns of interaction between their trajectories, further revealing the dynamic development relationship between variables. Based on the existing literature and theories, this study has a few overall hypotheses: (1) middle-aged and elderly activity participation shows a nonlinear downward trend with age, (2) middle-aged and elderly depression shows a nonlinear upward trend with age, (3) the activity participation of middle-aged and older adults predicts their depression during the same period, (4) the initial level and rate of change of activity participation in middle-aged and older adults could predict the development rate of the depression trajectory, and (5) the middle-aged and older adults’ early activity participation at 4 points in time predicts later depression.

## Methods

### Sample and Data Collection

Data were obtained from CHARLS. Our national baseline survey was launched in 2011, covering 150 county-level units, 450 village-level units, and about 17,000 people in 10,000 households, and these samples were tracked every 2-3 years thereafter. More details about CHARLS can be found in previous publications [28]. This database uses data from 4 waves in 2011 (wave I), 2013 (wave II), 2015 (wave III), and 2018 (wave IV), excluding respondents younger than 45 years old.

The detailed sample-screening process is shown in Multimedia Appendix 1. This study first focused on all respondents in wave I. Since the purpose of the study was to study the relationship between activity participation and depression, plus with the control of relevant time-varying and invariant variables, 3908 respondents of the final wave I were excluded from our study. The specific reasons were as follows: they were younger than 45 years or their age was not recorded (n=367, 9.4%); they had self-reported memory-related disorders and brain damage, intellectual disability, missing data on brain damage, or intellectual disability at baseline (n= 528, 13.5%); they had no baseline data on depressive symptoms (n=2101, 53.7%); they had no activity participation data at baseline (n=1943, 49.7%); and they had missing values for relevant covariates (gender, marriage, education, self-rated health, chronic disease, smoking, drinking; n=1943, 49.7%). Next, this study performed the same first process on wave II, wave III, and wave IV data as wave I, and in the second step, the study excluded respondents who had not participated in all of them for 4 years. Finally, after these operations, individuals with missing values on any variable were excluded, and a total of 4818 valid individuals were included.

### Ethical Considerations

This study was organized by the National Development Institute of Peking University, complied with the ethical guidelines of the 1975 Declaration of Helsinki, and was approved by the Ethics Committee of Peking University (approval number: IRB00001052-13074), hosted by the National Development Institute of Peking University. The studies involving human participants were reviewed and approved by the Research Ethics Committees of Peking University (IRB00001052-11015). The patients/participants provided their written informed consent to participate in this study.

### Activity Participation

Combining the definitions of activity participation by previous scholars [29] and the characteristics of activity participation of Chinese seniors [30], this study believed that the activity participation of Chinese seniors can be divided into these categories: exercise activities, social activities, and interaction with friends. Based on the characteristics of CHARLS, this study further summarized the activity participation of middle-aged and older people as physical activity participation (participation in exercise-related activities) and social leisure activity participation (participation in activities related to socializing and friend-making).

In addition, previous physical activity participation has mostly been measured only in terms of intensity, duration, and days of physical activity participation [31,32]. To measure physical activity participation more comprehensively, based on previous studies and the HRQoL scale of CHARLS [6,33], this study chose to comprehensively measure the physical activity participation of middle-aged and older people (as shown in Multimedia Appendix 2) from the aspects of PF, role-body (RP), body pain (BP), general health (GH).

In this study, PF measured whether health conditions prevent normal physical activity participation, the physiological functioning dimension measures whether physical health problems lead to limited participation in physical activity, the somatic pain dimension measures BP and its impact on participation in daily physical activities, and the overall health dimension measures their own evaluation of health status and trends [33]. In terms of measuring social leisure activity participation, based on a previous study [30], 2 questions were asked (as shown in Multimedia Appendix 2): What social activities have been carried out in the past month? What is the frequency of participating in these activities?

This study rescored these 2 types of activity parameters in reverse, ensuring that higher scores indicated more active activity participation. Then, based on the scoring rules of the Medical Outcomes Study (MOS) 36-item Short-Form (SF-36) Health Survey scale, the raw scores of the 2 activities’ participation in their respective components were calculated. The conversion scores of daily physical activity participation and social leisure activity participation were calculated using the range method between 0 and 100. Finally, the Cronbach α coefficient was used to measure the reliability of the above method. The results showed that the Cronbach α value of both physical activity participation and social leisure activity participation was higher than 0.6; hence, this scoring method is reliable [6,33].

### Depression

Depression was screened using the Chinese version of the Center for Epidemiologic Study Depression Scale (CES-D), with 10 short-form scale items [33], and results illustrated the magnitude of depression. The CES-D in CHARLS is a simplified version of the depression scale developed by Radolff at the National Institute of Mental Health [34]. The CES-D consists of 10 questions asking respondents about depression experienced 1 week before, and existing research has demonstrated its applicability in middle-aged and older Chinese populations [35]. Of these 10 questions, this study reverse-coded questions 5 and 8, and the total score was the sum of all questions. Each question is scored on a 4-point scale, ranging from 0 to 3, and the total score ranges from 0 to 30; therefore, the higher the score, the more severe the depression [36].

### Control Variable

Age, gender, education level, marital status, chronic disease, smoking, drinking, self-reported health, and number of chronic diseases were included as covariates in the analysis. The results of the 4 surveys of these variables were sorted out. This study took the baseline measurement results of gender and education level as time-invariant variables, leaving the remaining variables as time-varying variables. The education level was divided into “no formal education,” “primary school,” “junior high school,” and “high school education or above” [23,30]. Marital status included “married” and “other.” Self-perceived health status was reported as “very good,” “good,” “fair,” “poor,” or “bad.” The current smoking and drinking status was assessed by self-reporting based on the questions “Are you currently smoking?” and “Are you currently drinking?” Chronic diseases were all self-reported and marked as 0, 1, or at least 2 by the number of chronic diseases.

### Statistical Analysis

Descriptive analysis was performed using IBM SPSS (version 24.0) to determine the basic characteristics of all respondents.

**Figure 1:**
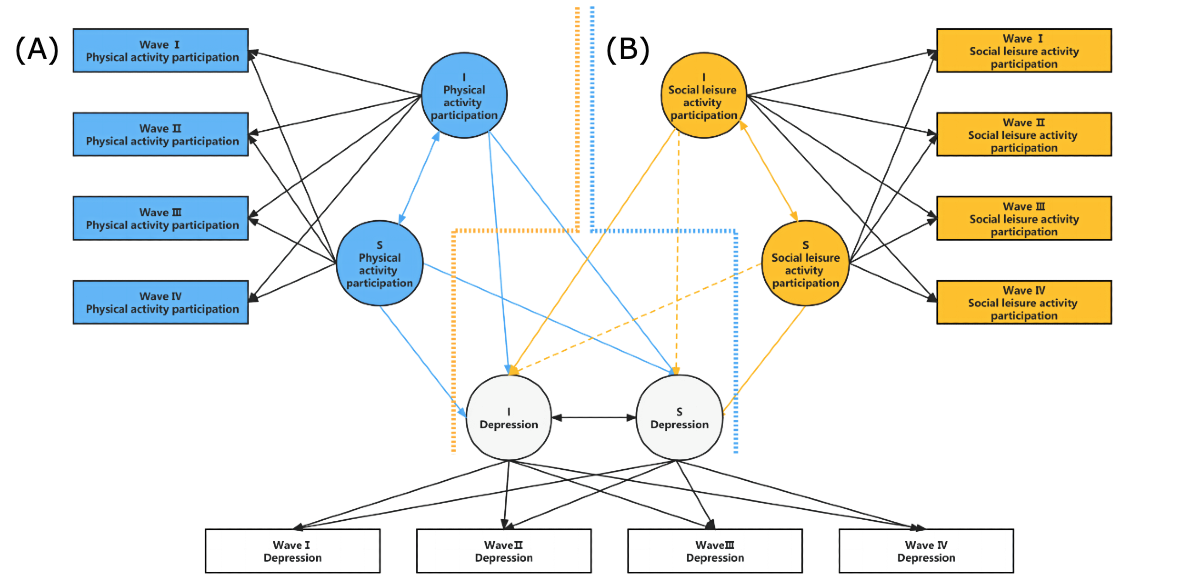
(A) LGCM of activity participation and depression. (B) LGCM of social leisure activity participation and depression. The activity participation intercept, activity participation slope, depression intercept, and depression slope were regressed on the covariates simultaneously. Valid paths and nonsalient paths are plotted as solid and dashed lines, respectively. I: intercept; LGCM: latent growth curve model; S: slope.

**Figure 2:**
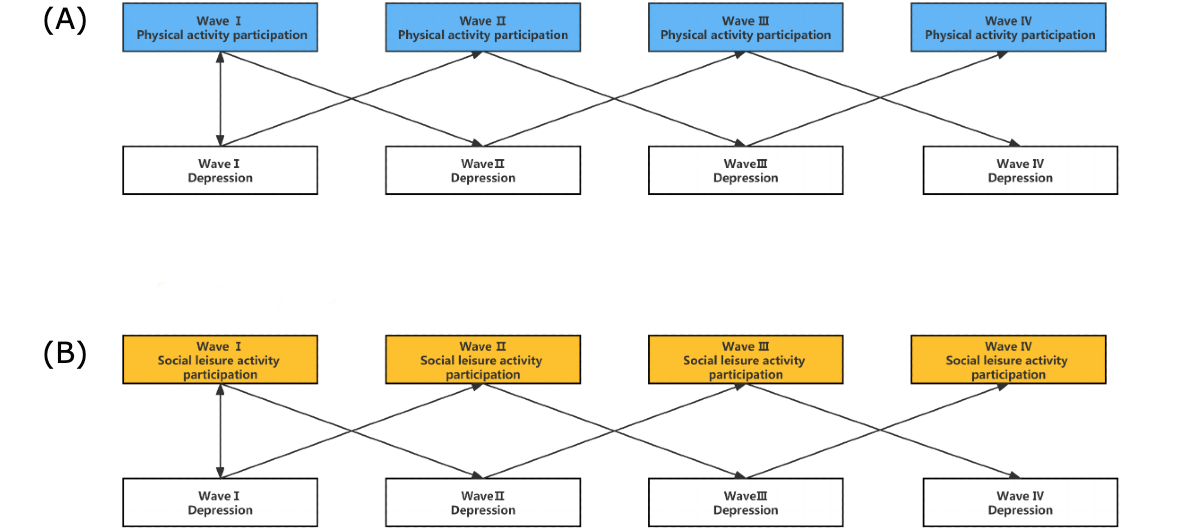
(A) Cross-lag model of physical activity participation and depression. (B) Cross-lag model of social leisure activity participation and depression. Note: activity participation and depression in all 4 waves were regressed on the covariates simultaneously.

The association of activity participation and depression was explored using Mplus (version 8.0). First, to study the respective trends in activity participation and depression in the middle-aged and older populations, an unconditional linear growth model and an unconditional quadratic growth model were constructed (see Multimedia Appendices 3 and 4). Second, a growth model with time-invariant and time-varying variables was constructed to study the interrelationship between depression and physical and leisure activities. Participation and depression were added to the model as time-varying variables, mainly looking at the relationship between activity participation and depression at the same time. Next, to avoid measurement errors and more accurately examine the relationship between depression and physical and social activities, a parallel latent growth model was developed (see Figure 1). Finally, after studying the dynamic properties of the variables using this model, the main lag relationship between depression and physical and social activities in the older population was further investigated using a cross-lag model (see Figure 2).

The hypothetical model fit was tested using multiple fit indices, including the standardized root mean square residual (SRMR), the root mean square error of approximation (RMSEA), the comparative fit index (CFI), and the Tucker-Lewis index (TLI). Specifically, if SRMR≤0.08, the model is acceptable; if RMSEA≤0.08, the model is acceptable. For CFI and TLI, values >0.90 are considered acceptable [37].

## Results

### Common Method Bias

The Harman single-factor test is a diagnostic technique to evaluate the severity of common method bias. In this study, the Harman single-factor test was performed on all the data from the 4 surveys. The test results showed that in these 4 surveys, the variance explained by the first factor was 21.7%, which is significantly less than 40.0%. This suggests that there is no common methodological bias in this study [38].

### Descriptive Statistics

Table 1 shows the descriptive statistics of all variables at baseline. The results showed that from 2011 to 2018, the average values of physical activity participation, social leisure activity participation, and depression ranged from 76.98 (SD 15.16) to 83.95 (SD 5.72), from 7.43 (SD 8.67) to 9.25 (SD 10.16), and from 7.61 (SD 5.72) to 8.82 (SD 6.51), respectively, respectively. Multimedia Appendices 5 and 6 are a clearer demonstration of the scores of and linear trends in depression, physical activity participation, and social leisure activity participation among middle-aged and older people during the 4 measurement periods. Multimedia Appendix 7 shows the binary correlations of key variables across 4 surveys. The results showed a negative correlation between physical activity participation, social leisure activity participation, and depression from 2011 to 2018. That is, the higher the scores of physical activity participation and social leisure activity participation, the lower the score of depression.

**Table 1:** Descriptive analysis of participants (N=4818).

Variable	Wave I, mean (SD)	Wave II, mean (SD)	Wave III, mean (SD)	Wave IV, mean (SD)
Age (years)	57.13 (7.95)	59.13 (7.94)	61.13 (7.95)	64.13 (7.95)
Marital status	1.36 (1.09)	1.41 (1.17)	1.45 (1.20)	1.64 (1.38)
Education level	3.65 (1.90)	2.06 (0.95)	2.05 (0.96)	1.95 (0.91)
Self-reported health	3.41 (0.10)	2.60 (1.01)	3.41 (1.06)	2.99 (1.01)
Chronic disease	1.31 (1.36)	1.58 (1.50)	1.79 (1.80)	2.37 (1.92)
Smoking	0.32 (0.47)	0.2 (0.40)	0.28 (0.45)	0.27 (0.44)
Drinking	0.35 (0.48)	0.29 (0.46)	0.37 (0.48)	0.34 (0.47)
Depression	7.93 (6.11)	7.61 (5.72)	7.75 (6.29)	8.62 (6.51)
Physical activity participation	83.95 (5.72)	79.25 (11.06)	77.43 (13.93)	76.98 (15.16)
Social leisure activity participation	7.43 (8.67)	9.25 (10.16)	8.93 (10.56)	8.25 (10.20)

### Developmental Trajectories of Depression Levels Among Seniors

To investigate the potential relationship between depressive symptoms and physical activity participation as well as social leisure activity participation, unconditional linear and unconditional quadratic latent growth curve models (LGCMs) were initially used to assess initial depression, physical activity participation, social leisure activity participation, and their trajectories over time in older adults. The results are shown in Multimedia Appendices 3 and 4, respectively. Multimedia Appendix 8 summarizes the fit indices for these models. Table 2 provides the within-subject mean for repeated measures (intercept/baseline), the between-subject variance in the intercept (intercept variance), the between-subject mean change (slope), the between-subject mean change (slope variance), and the quadratic slope variance and quadratic intercept variance.

With regard to the depression degree, both the linear growth model (model 1) and the quadratic growth model (model 2) of the depression degree were suitable (model 1: CFI=0.981, TCL=0.977, RMSEA=0.057, SRMR=0.031; model 2: CFI=1.000, TCL=1.001, RMSEA=1.001, SRMR=0.013). Although the fitness was acceptable, the quadratic slope variance of depression in the quadratic growth model of depression was not statistically significant (B=0.011, *P*=.045), as shown in Table 2. Therefore, the study chose to analyze the data from model 1. It was found that the variance estimates of the intercept factor and slope factor of the depression degree were 18.705 (*P*<.001) and 0.163 *P*<.001), respectively, indicating that there were significant interindividual differences in initial levels and growth rates. In addition, the annual mean slope of the depression level was 0.110 (SD 0.013, *P*<.001), meaning the depression level increased linearly during the 4 survey periods.

**Table 2:** Intercept and slope estimates for unconditional linear and nonlinear latent growth models.

Trajectory and models		Intercept (SE)	Intercept variance (SE)	Slope (SE)	Slope variance (SE)	Quadratic (SE)	Quadratic variance (SE)
**Trajectory of depression**							
	Model 1	7.595 (0.079)^a^	18.705 (0.653)^a^	0.110 (0.013)^a^	0.163 (0.026)^a^	N/A^b^	N/A
	Model 2	7.910 (0.086)^a^	16.305 (1.340)^a^	–0.243 (0.042)^a^	–0.074 (0.379)	0.049 (0.006)^a^	0.011 (0.005)
**Trajectory of physical activity participation**							
	Model 3	82.620 (0.112)^a^	17.351 (1.377)^a^	–0.913 (0.032)^a^	2.557 (0.132)^a^	N/A	N/A
	Model 4	83.817 (0.113)^a^	1.673 (3.334)^a^	–2.594 (0.090)^a^	9.874 (1.240)^a^	0.234 (0.011)^a^	0.046 (0.023)
**Trajectory of physical activity participation**							
	Model 5	7.848 (0.121)^a^	37.665 (1.575)^a^	0.134 (0.022)^a^	0.592 (0.077)^a^	N/A	N/A
	Model 6	7.265 (0.124)^a^	34.239 (3.580)^a^	1.028 (0.073)^a^	3.655 (1.058)^a^	–0.128 (0.010)^a^	0.049 (0.017)^a^

^a^The corresponding variable was statistically significant.

^b^N/A: not applicable.

### Developmental Trajectories of Physical Activity Participation and Leisure Activity Participation Among Older Adults

In terms of physical activity, the quadratic growth model (model 4) for their latent growth was better than the linear growth model (model 3); see Multimedia Appendix 8. With regard to the quadratic growth model for physical activity participation, the quadratic slope variance of physical activity participation was 0.046 (*P*=.048); see Table 2. Model 4 data were used as a result. Specifically, the initial level (intercept) of physical activity participation was 83.817 (*P*<.001). Physical activity participation decreased over the 4 test periods (slope=–2.594, *P*<.001), and the rate of decline increased year by year (curve slope=0.234, *P*<.001), indicating a nonlinear decline in physical activity participation over the 4 test periods. In addition, the estimated variances of the intercept factor and slope factor for physical activity participation were 1.673 and 9.874, respectively, and the variance of the slope factor was significant at the 0.001 level, indicating interindividual differences in growth rates.

Based on the fitting degree of the latent growth model of social leisure activity participation, the fitting degree of the quadratic growth model (model 6) of social leisure activity participation was better than that of the linear growth model (model 5) of social leisure activity participation (see Multimedia Appendix 8, Table S3). In addition, the quadratic slope variance of social leisure activity participation was 0.049 (*P*<.001); see Table 2. Therefore, model 6 data were used for this study. Specifically, the initial level of social leisure activity participation (intercept) was 7.265 (*P*<.001). The slope of social leisure activity participation during the 4 survey periods was 1.028 (*P*<.001), indicating that social leisure activity participation showed a linear upward trend in the 4 test periods (slope=1.028, *P*<.001), but the rate of increase decreased year by year (curve slope=–0.128, *P*<.001). In addition, the estimated variances of the intercept factor and the slope factor of social leisure activity participation were 34.239 (*P*<.001) and 3.655 (*P*<.001), respectively, indicating that there were significant interindividual differences in the initial level and growth rate.

### Relationship Between Physical Activity Participation and Social Leisure Activity Ability and Depression

To explore the potential relationship between the depression level and participation in physical and social leisure activities, a model was built with time-invariant and time-varying variables. This model examined whether taking physical activity participation and social leisure activity participation as time-varying variables has an effect on the depression level and whether the depression level is time-varying on physical activity participation when these endogenous variables are considered at the same time. The results of the model are shown in Table 3.

The results showed that at any time point, the lower the physical activity participation and social leisure activity participation, the higher the degree of depression, which means that physical activity participation and social leisure activity participation did affect the depression level of middle-aged and older people at corresponding time points, and based on the results, physical activity participation has a greater effect on depression than social leisure activity participation.

In addition, the effect of depression level on physical activity participation and social leisure activity participation was also considered. The results showed that at any time point, the higher the degree of depression, the lower the ability to participate in physical and social leisure activities, which means that the degree of depression does affect physical activity participation and social leisure activity participation for middle-aged and older people at corresponding time points.

**Table 3:** Standardized coefficients^a^ in LGCMs^b^ with depression, physical activity participation, and social leisure activity participation as time-varying variables.

Variables			Wave III depression		Wave II depression		Wave III depression		Wave IV epression
**Physical activity participation (depression**									
	Wave I physical activity participation (depression)	–0.167 (–0.024)		N/A^c^		N/A		N/A	
	Wave II physical activity participation (depression)	N/A		–0.284 (–0.05)		N/A		N/A	
	Wave III physical activity participation (depression)	N/A		N/A		–0.368 (–0.06)		N/A	
	Wave IV physical activity participation (depression)	N/A		N/A		N/A		–0.396 (–0.052)	
**Social leisure activity participation (depression)**									
	Wave I social leisure activity participation (depression)	–0.064 (–0.092)		N/A		N/A		N/A	
	Wave II social leisure activity participation (depression)	N/A		–0.058 (–0.058)		N/A		N/A	
	Wave III social leisure activity participation (depression)	N/A		N/A		–0.056 (–0.068)		N/A	
	Wave IV social leisure activity participation (depression)	N/A		N/A		N/A		–0.048 (–0.060)	

^a^All *P*<.001.

^b^LGCM: latent growth curve model.

^c^N/A: not applicable.

### Parallel LGCM

To avoid measurement errors and to more accurately examine whether depression is associated with physical activity participation and whether the trajectories change over time, a parallel LGCM was developed, as shown in Figure 1. This parallel LGCM examined the relationship between the intercepts and slopes of physical activity participation and social leisure activity participation and the intercepts and slopes of depression, and further examined the influence process between the 2 by setting regression equations between growth factors. This study, however, only focused on the interaction between the 2.

The parallel LGCM of physical activity participation for depression was well fitted (CFI=0.908, TLI=0.873, RMSEA=0.056). In Figure 1, the effective paths and nonsalient paths are drawn as solid and dashed lines, respectively. Detailed results are shown in Table 4. At the initial level, physical activity participation was negatively correlated with depression (r=–0.631, *P*<.001), that is, the worse the individual's initial physical activity participation, the higher the depression. Initial physical activity participation positively predicted the rate of change in depression (*β*=0.461, *P*<.001), indicating that individuals with higher initial physical activity participation scores have a higher rate of depression growth. Similarly, the slope of physical activity participation in the middle-aged and older populations affected not only the initial depression level (*β*=–0.261, *P*<.001) but also the rate of change in depression (*β*=–0.69, *P*<.001), indicating that the growth rate of physical activity participation suppresses the increase in the initial level of depression and its growth rate.

In addition, another parallel LGCM was built for social leisure activity participation and depression (see Table 4). The parallel LGCM fit well (CFI=0.951, TLI=0.933, RMSEA=0.033, RSMR=0.036). In Figure 1, the effective and nonsalient paths are plotted as solid and dashed lines, respectively. At the initial level, the degree of depression was negatively correlated with social leisure activity participation (r=–0.223, *P*<.001), indicating that the higher the individual's initial social leisure activity participation, the lower the depression. Initial social leisure activity participation did not predict the rate of change in depression (*β*=0.067, *P*=.159), indicating that initial social leisure activity participation is not associated with the rate of increase in depression. Similarly, although the slope of social leisure activity participation in the middle-aged and older populations did not affect the initial level of depression (*β*=0.08, *P*=.087), it did affect the rate of change in depression (*β*=–0.303, *P*<.001), indicating that the growth of social leisure activity participation suppresses the growth rate of depression.

**Table 4:** Results of LGCMs^a^ for activity participation and depression.

Path			Estimate (SE)		*P* value
**Physical activity participation → depression**					
	I^b^ physical activity participation → I depression	–0.631 (0.031)		<.001	
	S^c^ physical activity participation → I depression	–0.261 (0.025)		<.001	
	I physical activity participation → S depression	0.461 (0.065)		<.001	
	S physical activity participation → S depression	–0.69 (0.061)		<.001	
**Social leisure activity participation → depression**					
	I social leisure activity participation → I depression	–0.23 (0.027)		<.001	
	S social leisure activity participation → I depression	0.08 (0.047)		.087	
	I social leisure activity participation → S depression	0.067 (0.047)		.159	
	S social leisure activity participation → S depression	–0.303 (0.084)		<.001	

^a^LGCM: latent growth curve model.

^b^I: intercept.

^c^S: slope.

### Cross-Lag Model

The dynamic characteristics of the variables were studied using the LGCM. To further investigate the main lag relationship between activity participation and depression in the middle-aged and older populations, cross-lag regression analysis was performed across 4 measures. Cross-lag regression analysis can reveal complex relationships between 2 variables. The autoregressive effect of each variable is controlled by setting a coefficient of stability, which is the best way to test for “pure” effects between variables and to understand to what extent one variable predicts the other. In addition, to achieve a more compact model, the crossing paths across the waves were restricted to achieve equality, as shown in Figure 2. This procedure sets autoregressive effects as fixed effects, which avoids convergence problems. The relationship between variables was not considered stable across time, because only the lag effect was set as a random effect.

The results are shown in Table 5. Both activity participation and depression maintained high stability across the 4 measurements. Physical activity participation in the older population in wave I significantly and negatively predicted depression in wave II (*β*=–0.125, *P*<.001). Physical activity participation in the middle-aged and older populations in wave II significantly and negatively predicted depression in wave III (*β*=–0.216, *P*<.001). Physical activity participation in the older population in wave III significantly and positively predicted depression in wave IV (*β*=–0.233, *P*<.001). Likewise, prior depression also had a significant negative impact on later physical activity participation.

**Table 5:** Cross-lag regression path coefficients.^a^

Regression path		Wave I to wave II	Wave II to wave III	Wave III to wave IV
**Physical activity participation and depression**				
	Physical activity participation → depression	–0.125	–0.216	–0.233
	Depression → physical activity participation	–0.874	–0.737	–0.685
	Depression → depression	0.456	0.414	0.414
	Physical activity participation → physical activity participation	0.09	–0.049	–0.049
**Social leisure activity participation and depression**				
	Social leisure activity participation → depression	–0.04	–0.045	–0.044
	Depression → social leisure activity participation	–0.058	–0.054	–0.058
	Depression → depression	0.515	0.475	0.481
	Social leisure activity participation → social leisure activity participation	0.399	0.430	0.446

^a^All *P*<.001.

Similar results were shown in the cross-lag model of social leisure activity participation and depression as in the cross-lag model of physical activity participation and depression. The social leisure activity participation of the older population in wave I significantly and negatively predicted depression in wave II (*β*=–0.04, *P*<.001). Social leisure activity participation in the middle-aged and older populations in wave II significantly and negatively predicted depression in wave III (*β*=–0.045, *P*<.001). The social leisure activity participation of the older population in wave III significantly and predicted depression in wave IV (*β*=–0.044, *P*<.001). Similarly, previous depression also had a significant negative impact on later social leisure activity participation.

## Discussion

### Principal Results

Based on a CHARLS survey spanning 7 years, this study found that activity participation has a long-term effect on depression. First, physical activity participation decreased significantly between 2011 and 2018 among middle-aged and older Chinese populations, suggesting that the physical activity participation of middle-aged and older adults decreases with age [14,18]. Studies have shown that chronic diseases related to aging can lead to the risk of arthritis and disability and that impairment of activities of daily living gradually increase with age [39,40], which in turn leads to a lifestyle change in middle-aged and older people. A series of chain reactions end up with a reduction in physical activity participation [41]. Life cycle theory can explain this change well. This study selected respondents over the age of 45 years. As the survey continued, the life cycle of these respondents gradually entered a decline period, so their PFs also began to decline. We can even speculate that this phenomenon might be more pronounced if the initial age restriction is controlled to over 60 years [14]. However, it is worth mentioning that it is precisely because of our selection of the age of ≥45 years (the average age in wave I was 57 years) that as these people get older, they start to withdraw from work (the Chinese government stipulates that 60 years is the retirement age for citizens) and have more time for social leisure activity. Therefore , there was a significant increase in leisure activity participation from wave I to wave II. However, after wave II, most of them (the average age in wave II was 60 years) also entered old age, and social leisure activity began to decline continuously, which is also consistent with previous studies [14]. Finally, based on the analysis of the trajectory of depression, the level of depression in middle-aged and older people in China increased significantly between 2011 and 2018, and updated evidence was provided again to prove that the trajectory of depression is affected by age [8,23,42].

Second, the study found a horizontal correlation between activity participation and depression within the same period and further demonstrated that this relationship persisted during different periods. Specifically, models incorporating time-varying and invariant variables proved that between 2011 and 2018, activity participation in middle-aged and older adults significantly predicted depression in the same period and that depression in middle-aged and older adults also significantly predicted participation in activities in the same period. This suggests that the trajectory of depression is affected by activity participation, and the developmental trajectory of activity participation is similarly affected by depression [42-44]. The behavioral theory of depression and BA theory can successfully explain the mutual influence of activity participation on depression. The behavioral theory of depression states that low-response-conditioned positive reinforcement (RCPR) rates can directly trigger depression, so depression can be improved by re-establishing RCPR rates through altering activity participation and other activities that make individuals feel pleasant [45] and that improvements in depression in turn will lead to greater activity participation [46]. Previous studies have also found that the activity participation of middle-aged and older people may be plagued by poor physical and mental health (eg, depression, anxiety) [47] and that poor mental health and related symptoms (eg, irritability) result in reducing activity participation through perceptions of stress, perceptions of health, and some physical symptoms [27].

Additionally, a parallel LGCM showed that the higher the initial level of activity participation, the slower the rise in depression, which is consistent with previous analyses that showed that activity participation is a protective factor for depression among older adults [48,49]. Active participation in various activities as an active lifestyle attitude in middle-aged and older people can inhibit the increase in depression [50]. Moreover, the parallel LGCM also found that the slope of activity participation was significantly correlated with the slope of depression, suggesting that the rate of decline in activity participation among older adults could predict the rate of decline in depression, meaning that the faster the decline in activity participation, the sooner the depression rate increase. In addition, our study suggests that the development of depression partly influences the development of activity participation. One possible explanation is that depression seriously affects the physical health of patients [51], and physical health is directly related to the activity participation of middle-aged and elderly people [52].

Finally, the study performed a cross-lag regression analysis on activity participation and depression in middle-aged and elderly populations to observe the time series between activity participation and depression. Results showed that activity participation in middle-aged and older people can positively predict subsequent depression and that depression can also predict subsequent activity participation; however, no 1-way time series of activity participation and depression was found. This result further proves the bidirectional correlation between activity participation and depression in middle-aged and older people [53].

### Limitations

This study has some limitations. First, the participation data of this research activity came from the subjective answers of middle-aged and older people. As much as the study team tried to control some biases caused by subjective answers, such as excluding respondents with cognitive impairment, intellectual disability, and memory problems from the study, there would still be problems posed by subjective answers. Second, physical activity participation and social leisure activity participation were categorized to represent the activity participation of middle-aged and older people. The frequency of participation and the quality of activities were included in the selection of indicators; however, with regard to the different types of activity participation, the classification is still not detailed. For future studies, a detailed division of different types of activity participation should be made to compare the effects of different types of activity participation on the physical and mental health of middle-aged and older people. Finally, the focus of the study was the effect of activity participation on depression. Although the study found that the development of depression also affects the development of activity participation, it did not focus on explaining this relationship, and future research should pay more attention to depression’s impact on activity participation.

### Conclusion

Study results support a bidirectional association between activity participation and depression. Activity participation affects not only the initial level of depression but also the trend in changes in depression. In the future, more attention should be paid to depression in middle-aged and older people with less active participation. The interconnectedness of early activity participation and subsequent depression is noteworthy and may have implications for public health. Overall, this study highlights the relationship between activity participation and depression level changes, bringing positive implications for preventing depression aggravation in later life.

## Appendix

Multimedia Appendix 1 Flowchart of study respondents.

Multimedia Appendix 2 HRQoL variable selection. HRQoL: health-related quality of life.

Multimedia Appendix 3 Unconditional linear LGCM. i: intercept; LGCM: latent growth curve model; s: slope.

Multimedia Appendix 4 Unconditional nonlinear LGCM. i: intercept; LGCM: latent growth curve model; s: slope.

Multimedia Appendix 5 Change trajectory of depression.

Multimedia Appendix 6 Change trajectory of physical activity participation and social leisure activity participation.

Multimedia Appendix 7 Correlation coefficient matrix.

Multimedia Appendix 8 Fit statistics for LGCMs. LGCM: latent growth curve model.

## Abbreviations

BA: behavioral activation
BP: body pain
CES-D: Center for Epidemiologic Study Depression Scale
CFI: comparative fit index
CHARLS: China Health and Retirement Longitudinal Study
HRQoL: health-related quality of life
LGCM: latent growth curve model
PF: physical function
RCPR: response-conditioned positive reinforcement
RMSEA: root mean square error of approximation
SRMR: standardized root mean square residual
TLI: Tucker-Lewis index

## Data Availability

The data sets generated and analyzed during the study are available from the corresponding author upon reasonable request.
