# Supplementary figures and images for: The Effect of Activity Participation in Middle-Aged and Older People on the Trajectory of Depression in Later Life: National Cohort Study
Source: JMIR Public Health Surveill. 2023 Mar 23;9:e44682. doi: 10.2196/44682 (PMC10131905; doi:10.2196/44682)

**Multimedia Appendix 1.**

**Figure S1.** The flow chart of study respondent.


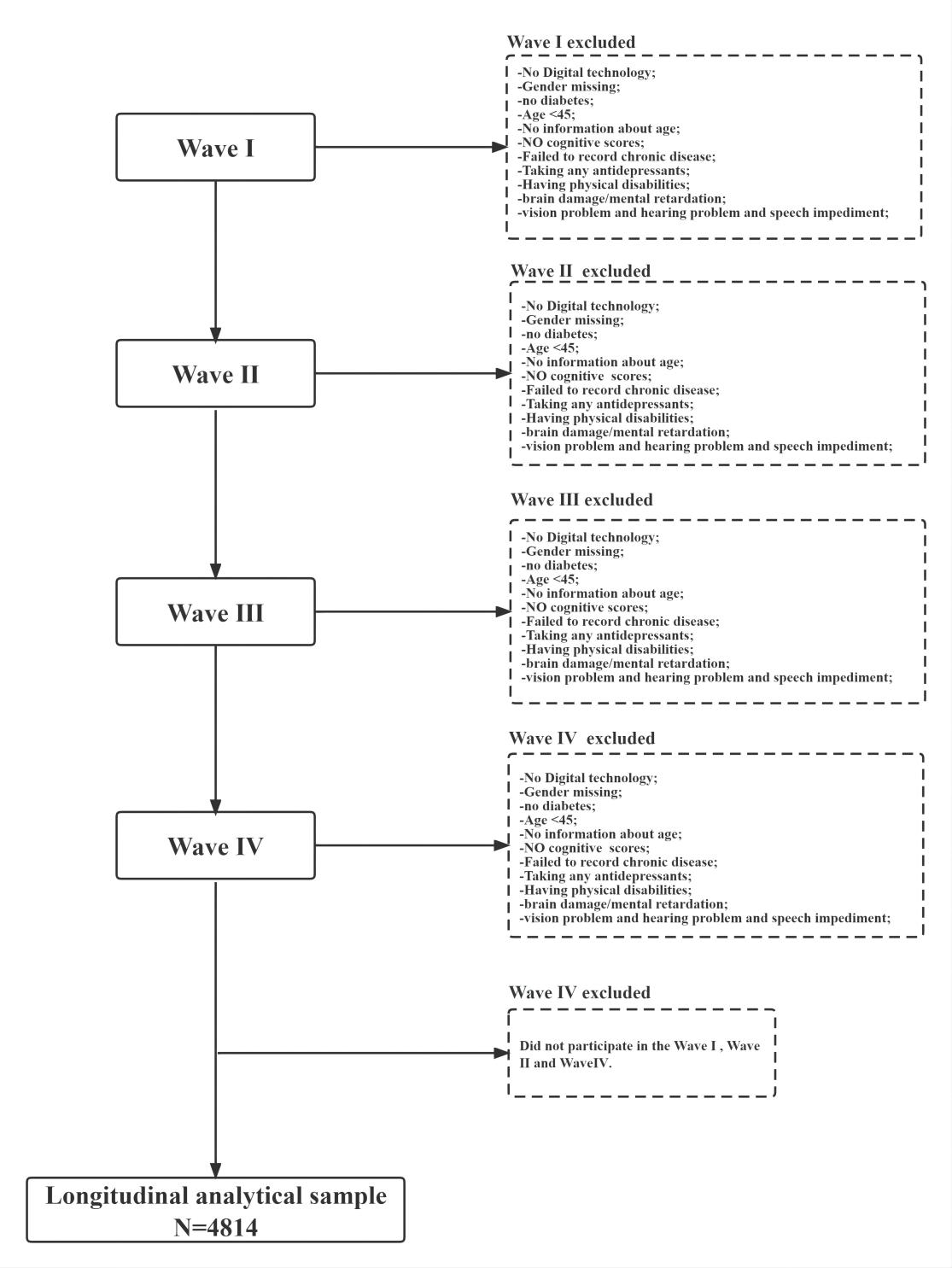

Supplement: Multimedia Appendix 1 [file publichealth_v9i1e44682_app1.docx]

**Multimedia Appendix 5.**

**Figure S4.** Change trajectory of Depression.


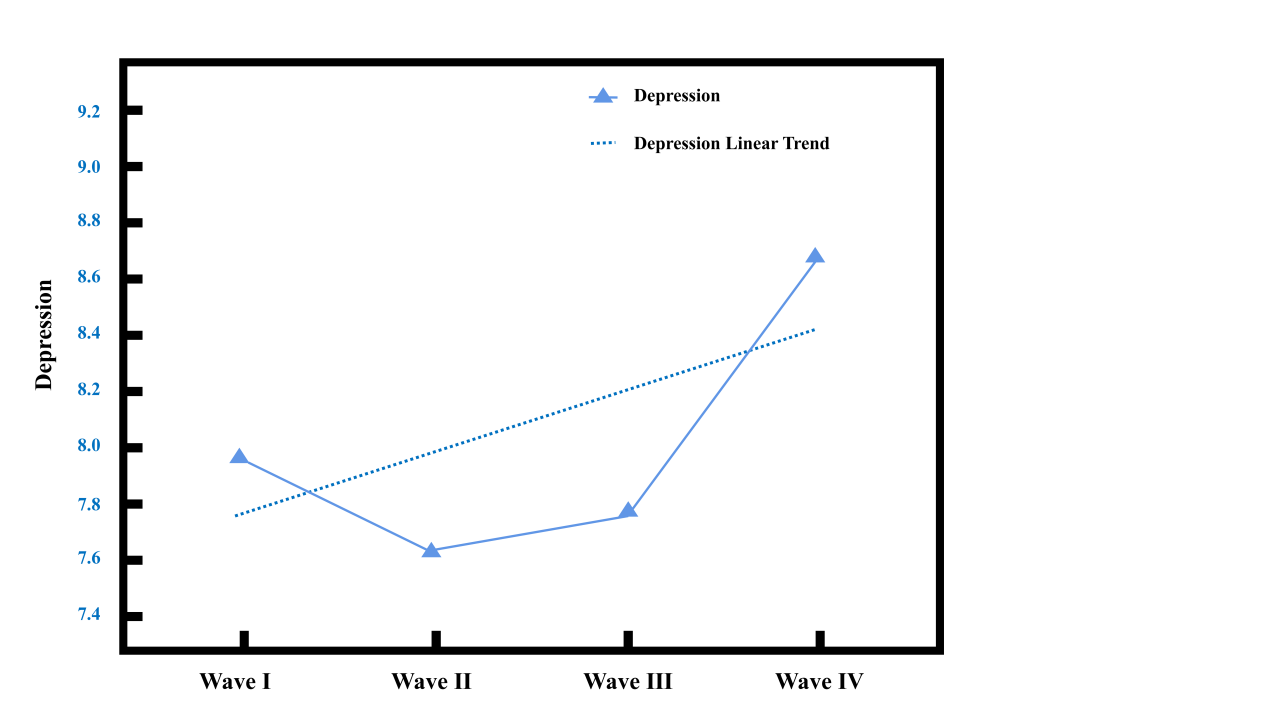

Supplement: Multimedia Appendix 5 [file publichealth_v9i1e44682_app5.docx]
