# Supplementary material for: The Effect of Activity Participation in Middle-Aged and Older People on the Trajectory of Depression in Later Life: National Cohort Study
Source: JMIR Public Health Surveill. 2023 Mar 23;9:e44682. doi: 10.2196/44682 (PMC10131905; doi:10.2196/44682)
Supplement: Multimedia Appendix 2 [file publichealth_v9i1e44682_app2.docx]

**Multimedia Appendix 2.**

**Table S1.** HRQoL Variable selection.

| **Variable** | | **CHARLS validity** |
| --- | --- | --- |
| physical activity participation | PF | db001 db002 db003 db004 db005 db006 db007 db008 db009 |
|  | RP | db016 db017 db018 db019 db020 |
|  | BP | da041 da042s1 da042s2 da042s3 da042s4 da042s5 da042s6 da042s7 da042s8 da042s9 da042s10 da042s11 da042s12 da042s13 da042s14 da042s15 |
|  | GH | da001 da002 |
| social leisure activity participation | | da056s1 da056s2 da056s3 da056s4 da056s5 da056s6 da056s7 da056s8 da056s9 da056s10 da056s11 da056s12; da057s1 da057s2 da057s3 da057s4 da057s5 da057s6 da057s7 da057s8 da057s9 da057s10 da057s11 |

**Note:** HRQoL: health-related quality of life; CHARLS: the China Health and Retirement Longitudinal Study.
