# Supplementary material for: The Effect of Activity Participation in Middle-Aged and Older People on the Trajectory of Depression in Later Life: National Cohort Study
Source: JMIR Public Health Surveill. 2023 Mar 23;9:e44682. doi: 10.2196/44682 (PMC10131905; doi:10.2196/44682)

**Multimedia Appendix 4.**

**Figure S3.** Unconditional Nonlinear Latent Growth Curve Model. Note: i denote intercept; s denote slope.


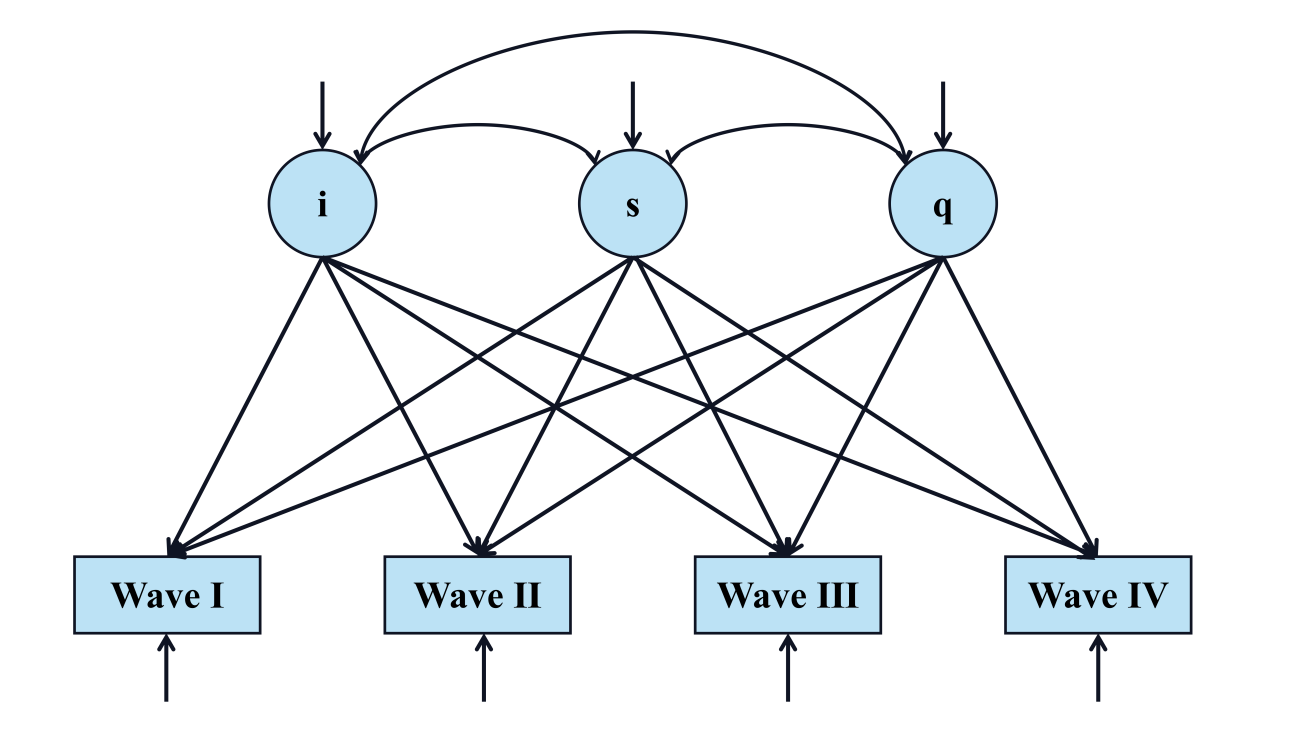

Supplement: Multimedia Appendix 4 [file publichealth_v9i1e44682_app4.docx]
