# Supplementary material for: The Effect of Activity Participation in Middle-Aged and Older People on the Trajectory of Depression in Later Life: National Cohort Study
Source: JMIR Public Health Surveill. 2023 Mar 23;9:e44682. doi: 10.2196/44682 (PMC10131905; doi:10.2196/44682)

**Multimedia Appendix 6.**

**Figure S5.** Change trajectory of physical activity participation and social leisure activity participation..


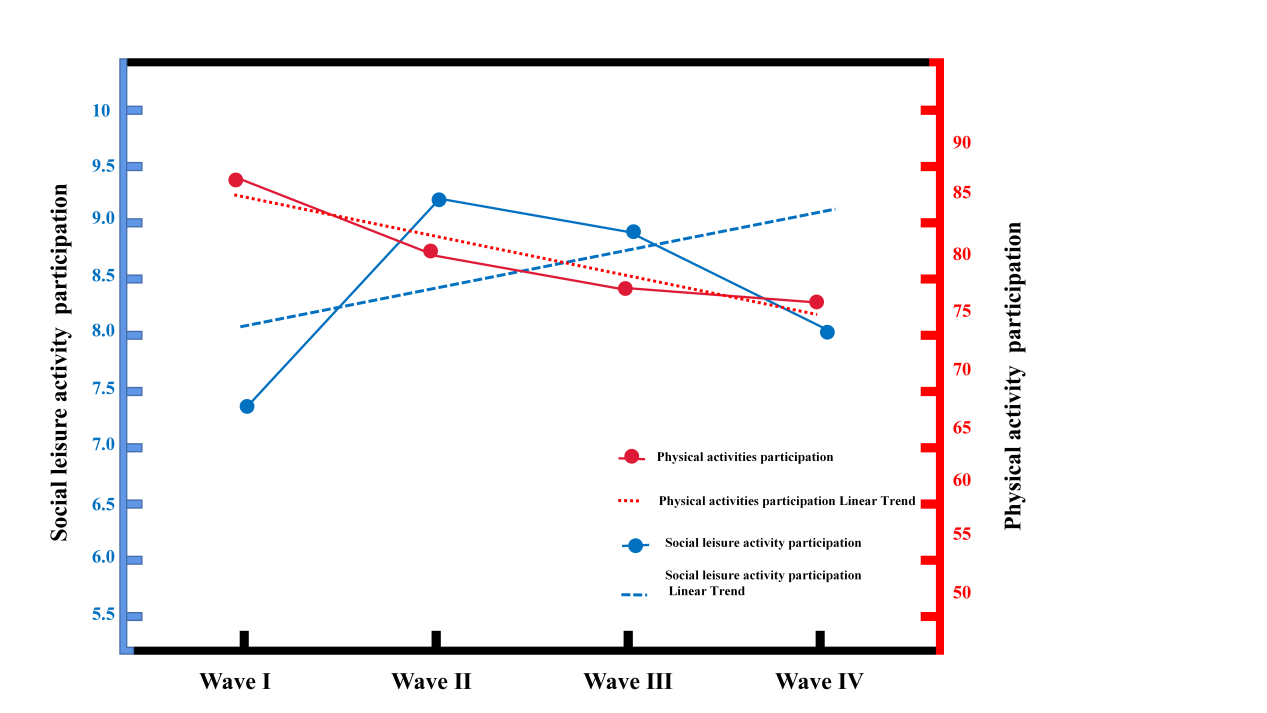

Supplement: Multimedia Appendix 6 [file publichealth_v9i1e44682_app6.docx]
