# Supplementary material for: The Effect of Activity Participation in Middle-Aged and Older People on the Trajectory of Depression in Later Life: National Cohort Study
Source: JMIR Public Health Surveill. 2023 Mar 23;9:e44682. doi: 10.2196/44682 (PMC10131905; doi:10.2196/44682)
Supplement: Multimedia Appendix 7 [file publichealth_v9i1e44682_app7.docx]

**Multimedia Appendix 7.**

**Table S2.** Correlation coefficient matrix (n=4818).

|  | Wave Ⅰ P | Wave Ⅱ P | Wave Ⅲ P | Wave Ⅳ P | Wave Ⅰ S | Wave Ⅱ S | Wave Ⅲ S | Wave Ⅳ S | Wave Ⅰ D | Wave Ⅱ D | Wave Ⅲ D | Wave Ⅳ D |
| --- | --- | --- | --- | --- | --- | --- | --- | --- | --- | --- | --- | --- |
| Wave Ⅰ P | 1 |  |  |  |  |  |  |  |  |  |  |  |
| Wave Ⅱ P | 0.24** | 1 |  |  |  |  |  |  |  |  |  |  |
| Wave Ⅲ P | 0.22** | 0.39** | 1 |  |  |  |  |  |  |  |  |  |
| Wave Ⅳ P | 0.16** | 0.51** | 0.49** | 1 |  |  |  |  |  |  |  |  |
| Wave Ⅰ S | 0.03* | 0.25** | 0.10** | 0.16** | 1 |  |  |  |  |  |  |  |
| Wave Ⅱ S | 0.06** | 0.16** | 0.12** | 0.27** | 0.41** | 1 |  |  |  |  |  |  |
| Wave Ⅲ S | 0.07** | 0.16** | 0.12** | 0.17** | 0.39** | 0.47** | 1 |  |  |  |  |  |
| Wave Ⅳ S | 0.04** | 0.14** | 0.13** | 0.15** | 0.33** | 0.40** | 0.45** | 1 |  |  |  |  |
| Wave Ⅰ D | -0.22** | -0.97** | -0.39** | -0.51** | -0.13** | -0.12** | -0.12** | -0.10** | 1 |  |  |  |
| Wave Ⅱ D | -0.14** | -0.50** | -0.47** | -0.96** | -0.10** | -0.13** | -0.11** | -0.10** | 0.52** | 1 |  |  |
| Wave Ⅲ D | -0.17** | -0.47** | -0.40** | -0.54** | -0.10** | -0.10** | -0.12** | -0.12** | 0.49** | 0.55** | 1 |  |
| Wave Ⅳ D | -0.14** | -0.43** | -0.35** | -0.49** | -0.10** | -0.11** | -0.13** | -.12** | 0.44** | 0.50** | 0.54** | 1 |

**Note:***p<0.05, **p<0.01; P: physical activity participation, S: social leisure activity participation, D: Depression.
