# Supplementary material for: The Effect of Activity Participation in Middle-Aged and Older People on the Trajectory of Depression in Later Life: National Cohort Study
Source: JMIR Public Health Surveill. 2023 Mar 23;9:e44682. doi: 10.2196/44682 (PMC10131905; doi:10.2196/44682)
Supplement: Multimedia Appendix 8 [file publichealth_v9i1e44682_app8.docx]

**Multimedia Appendix 8.**

**Table S3.** Fit statistics for latent growth curve models.

|  | Model | CFI^a^ | TLI^b^ | RMSEA^c^（90% CI） | SRMR^d^ |
| --- | --- | --- | --- | --- | --- |
| Depression | Model 1 | 0.984 | 0.981 | 0.062(0.051,0.073) | 0.031 |
|  | Model 2 | 1.000 | 1.001 | 0.000(0.000,0.032) | 0.001 |
| physical activity participation | Model 3 | 0.829 | 0.795 | 0.202(0.191,0.213) | 0.277 |
|  | Model 4 | 0.998 | 0.986 | 0.054(0.032,0.079) | 0.010 |
| social leisure activities participation | Model 5 | 0.921 | 0.905 | 0.112(0.102 0.123) | 0.048 |
|  | Model 6 | 0.992 | 0.95 | 0.081(0.058,0.106) | 0.016 |

^a^CFI: comparative fit index.

^b^TLI: Tucker-Lewis index.

^c^RMSEA: the root mean square error of approximation.

^d^SRMR: the standardized root mean square residual.
